# Self-assembled fibre optoelectronics with discrete translational symmetry

**DOI:** 10.1038/ncomms12807

**Published:** 2016-10-04

**Authors:** Michael Rein, Etgar Levy, Alexander Gumennik, Ayman F. Abouraddy, John Joannopoulos, Yoel Fink

**Affiliations:** 1 Department of Materials Science and Engineering, Massachusetts Institute of Technology, Cambridge, Massachusetts 02139, USA; 2 Research Laboratory of Electronics (RLE), Massachusetts Institute of Technology, Cambridge, Massachusetts 02139, USA; 3 Institute for Soldier Nanotechnologies, Massachusetts Institute of Technology, Cambridge, Massachusetts 02139, USA; 4 Department of Intelligent Systems Engineering, Indiana University Bloomington, Bloomington IN 47408-2664, USA; 5 Center for Research and Education in Optics and Lasers (CREOL), The College of Optics and Photonics, University of Central Florida, Orlando, Florida 32816, USA; 6 Department of Physics, Massachusetts Institute of Technology, Cambridge, Massachusetts 02139, USA

## Abstract

Fibres with electronic and photonic properties are essential building blocks for functional fabrics with system level attributes. The scalability of thermal fibre drawing approach offers access to large device quantities, while constraining the devices to be translational symmetric. Lifting this symmetry to create discrete devices in fibres will increase their utility. Here, we draw, from a macroscopic preform, fibres that have three parallel internal non-contacting continuous domains; a semiconducting glass between two conductors. We then heat the fibre and generate a capillary fluid instability, resulting in the selective transformation of the cylindrical semiconducting domain into discrete spheres while keeping the conductive domains unchanged. The cylindrical-to-spherical expansion bridges the continuous conducting domains to create ∼10^4^ self-assembled, electrically contacted and entirely packaged discrete spherical devices per metre of fibre. The photodetection and Mie resonance dependent response are measured by illuminating the fibre while connecting its ends to an electrical readout.

## Abstract (web summary)

The thermal-draw technique offers fibre devices with a multiplicity of geometries, but these are constrainted to being translationally symmetric. Here, the authors disrupt this symmetry by applying selective capillary instability, resulting in electrically connected spherical photodetecting elements.

## Graphical abstract

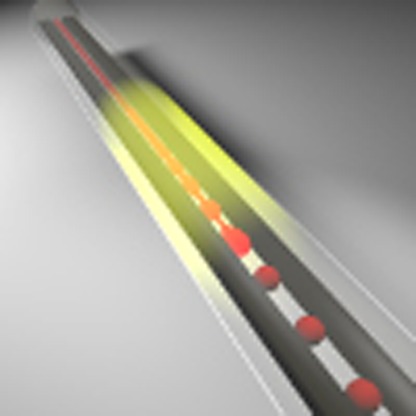

In recent years, multimaterial fibre devices with capabilities ranging from acoustic sensing to transduction^1^,^2^,^3^,^4^ to multimodal neural interfaces^5^ have emerged. These devices typically contain continuous conductive domains spanned by a material that responds to a stimulus or excitation. Drawn from a fluid state, the interfaces between the disparate fibre materials define a surface tension that renders the continuous internal domains thermodynamically unstable with respect to their discrete counterparts^6^,^7^.

A fibre with multiple continuous internal fluid domains of disparate sizes and composition will, in principle, undergo break-up and form parallel arrays of spheres, each with its own periodicity, as illustrated by Fig. 1a. However, since the kinetics of fluid instabilities are dictated not only by surface tension, but also by the dimensions of the domain and its viscosity, one can consider the possibility of designing a fibre where a selective break-up occurs targeting only specific domains, while keeping others continuous as illustrated in Fig. 1b. By doing so, one could maintain the electrical transport benefits of the continuous domains while taking advantage of discrete geometries that offer differentiated device attributes.

In the following, we report on thermal draw of a macroscopic preform, which results in a fibre that has three parallel internal non-contacting continuous domains; a semiconducting glass between two conductive polymers. We then heat the fibre and generate a capillary fluid instability, which results in the selective transformation of the cylindrical semiconducting domain into discrete spheres while keeping the conductive domains unchanged. The cylindrical-to-spherical lateral expansion bridges the continuous conducting buses to create ∼10^4^ self-assembled, electrically contacted and entirely packaged discrete spherical devices per meter of fibre. These results in a photodetecting fibre devices with spherical active components, whose Mie resonance dependent wavelength responsivity is measured.

## Results

### Principles of selective break-up

Fluid instability in fibres can be described by an instability model for a cylindrical thread of viscous liquid (core of the fibre) that is surrounded by another viscous liquid (the cladding of the fibre) as developed by Tomotika^8^. According to this model, a sinusoidal perturbation of a wavelength *λ* grows exponentially with a time constant *τ* given by


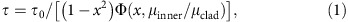


where *x*≡2*πa*/*λ*, *a* is the radius of the inner fluid, either the semiconducting core or the electrodes; 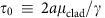, *μ*_clad_ and *μ*_inner_ are the viscosity of the cladding and the inner fibre component, respectively; *γ* is the interfacial surface tension between the two liquids, and Φ is a function defined in ref. 8, and displayed in Supplementary Note 1.

The fibre was designed such that at a temperature range of 200–300 °C, the capillary break-up timescale of the semiconducting core would occur over several minutes—an order of magnitude shorter timescale than that of the conducting domains.

**Figure 1:**
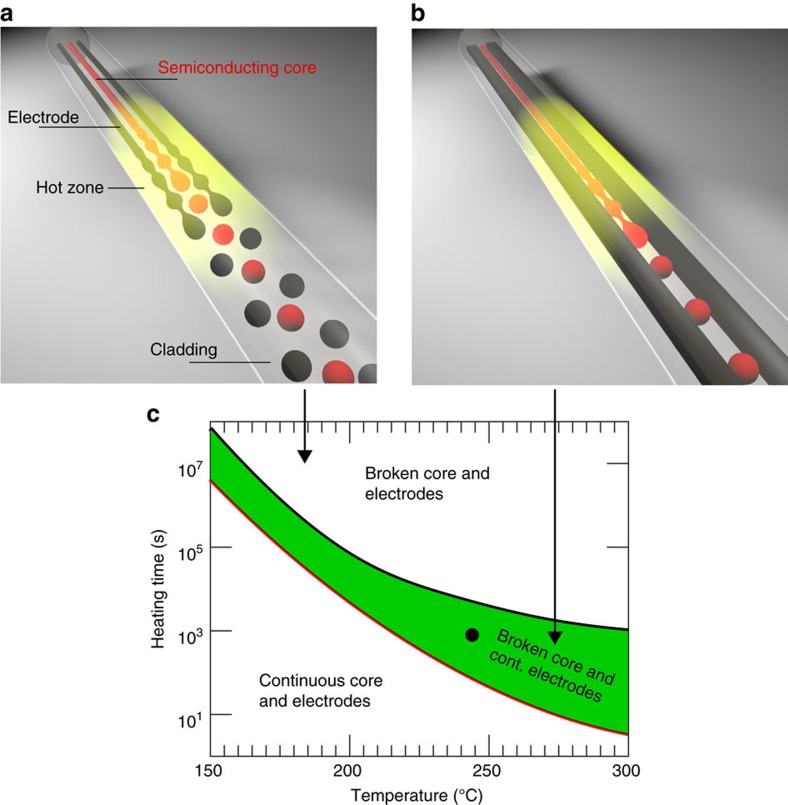
The approach towards selective break-up in multicore fibres. (**a**) Illustration of the break-up process of a fibre that contains three cores when the heating duration is longer than the capillary break time constant of the three cores. This case corresponds to the area marked as ‘Broken core and electrodes' in **c**. (**b**) Illustration of the selective break-up process. A semiconducting core fibre that is placed on a hot plate is heated (illustrated by the yellow area) and the inner core undergoes break-up while the electrodes stay continuous—forming self-assembled spherical photodetectors inside the fibre. This case corresponds to the area marked as ‘Broken core continuous electrodes' in **c**. (**c**) Fibre inner component ‘phase' diagram calculated for the semiconducting core (red) and electrodes (black). Long heating time of the fibre (post draw), at high temperature leads to electrode and core break-up. Lines correspond to the timescale of break-up (as defined by equation (1)). Selective break-up is achieved in the domain between the red and black curves (shaded green area). The conditions that were used in our case are denoted by a black circle. Calculation performed for a semiconducting core radius of 50 μm and electrode cross section of 90 × 190 μm.

With the above design criteria in mind, the inner core was chosen from equation (1) to be an amorphous semiconducting material—As_2_Se_5_. The electrodes were chosen to be a carbon black filled polyethylene matrix (CPE), with lateral dimensions twofold greater than the semiconducting core. Polycarbonate (PC) was chosen as the cladding material as it is perfectly transparent in the visible and near-infrared wavelength domain, and allows for *in-situ* optical observation of the break-up process. The surface tension between As_2_Se_5_ and PC is (ref. 9) 114 mJ m^−2^, which is significantly higher than that of CPE–PC interface^10^—30 mJ m^−2^. These surface tension and cross sectional dimension disparities yield electrode break-up timescales that are more than one order of magnitude longer than the core disintegration timescale as shown by the black and red lines in Fig. 1c. (This figure was derived from data shown in Supplementary Fig. 1 and Supplementary Table 1).

### Fibre draw

**Figure 2:**
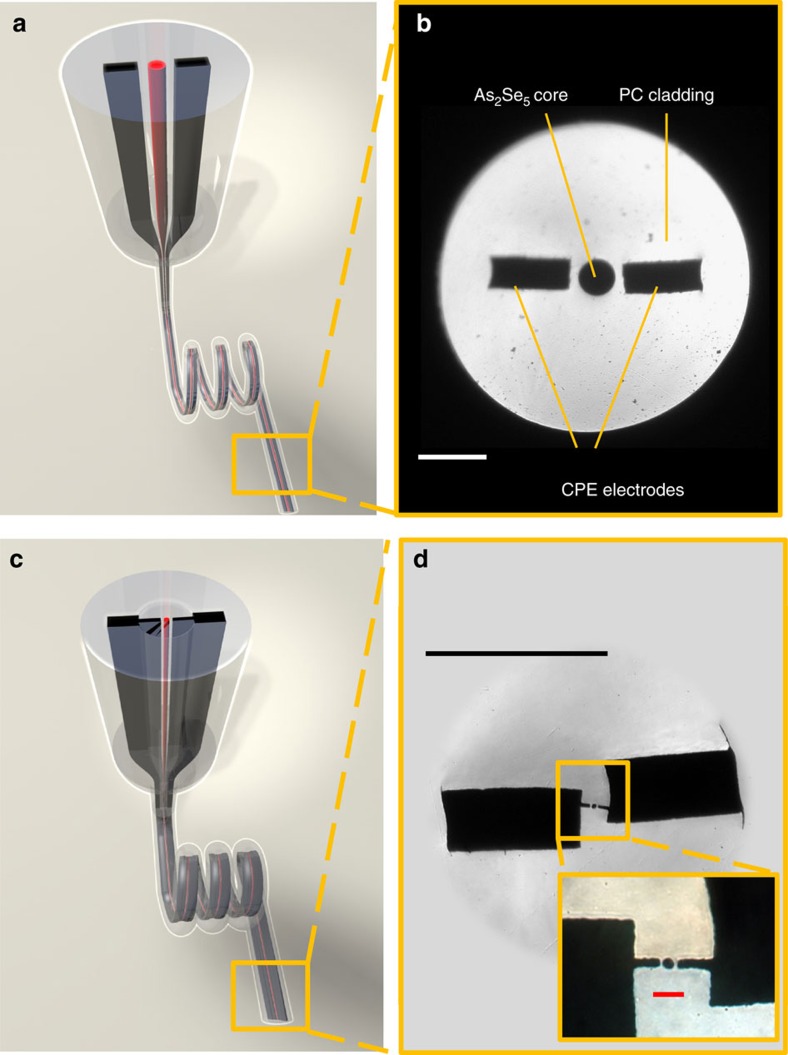
Fibre structure and draw. (**a**) Illustration of the fibre preform structure—chalcogenide glass (As_2_Se_5_) (red core) and two carbon black polyethylene composite (CPE) electrodes (black cores) are inserted into a polycarbonate (PC) cladding. The preform is scaled down in the drawing tower furnace into a fibre. (**b**) Optical micrograph of the cross section of the fibre shown in **a**. The semiconducting core has a radius of 50 μm. (Scale bar, 200 μm) (**c**) The fibre illustrated in **a** is inserted into another preform with extended electrodes to facilitate connection to the fibre after draw. This preform is drawn again in order to reduce the size of the chalcogenide core. (**d**) Optical micrograph of a redrawn fibre with extended electrodes and a chalcogenide core of 5 μm radius; Inset—optical micrograph of the active region of the redrawn fibre. (Top black scale bar, 100 μm; Bottom red scale bar, 20 μm).

The fibres were produced starting with a macroscopic preform, such that when scaled down through a thermal drawing method, results in a fibre of the desired cross sectional structure. Figure 2a,b demonstrate the process of the fibre draw, of a fiber that contains a central semiconducting core radius of 50 μm, flanked by two electrodes placed in the proximity of the core with a gap to be bridged by the spheres in the break-up process.

To avoid fibre shrinkage during the subsequent break-up, the draw was performed at relatively high temperatures (270−300 °C) to ensure a draw stress below 100 gr mm^−2^. It is worthwhile to note that the drawn structures do not undergo break-up during the draw process itself as the time the fibre spends in the hot zone of the furnace is much shorter than the timescale required for break-up.

To further reduce the radius of the semiconducting cores from 50 to 5 μm and 2.5 μm, the 50 μm core fibre was inserted into a core of subsequent preform made of PC and was redrawn. Extensions to the electrodes were added to the encapsulating preform at the redraw step to facilitate electrical interfacing to the redrawn fibre, as shown in Fig. 2c,d. This allowed to reduce the sphere diameter and hence to achieve spectrally resolvable resonant light interaction with the spheres. Generally, this approach allows forming electrical connection to a wide range of component sizes, demonstrating a hierarchical structure for electrical connection that produces a macroscopic electrical connection to microsphere arrays spanning three orders of magnitude of size difference.

### Inducing selective break-up

**Figure 3:**
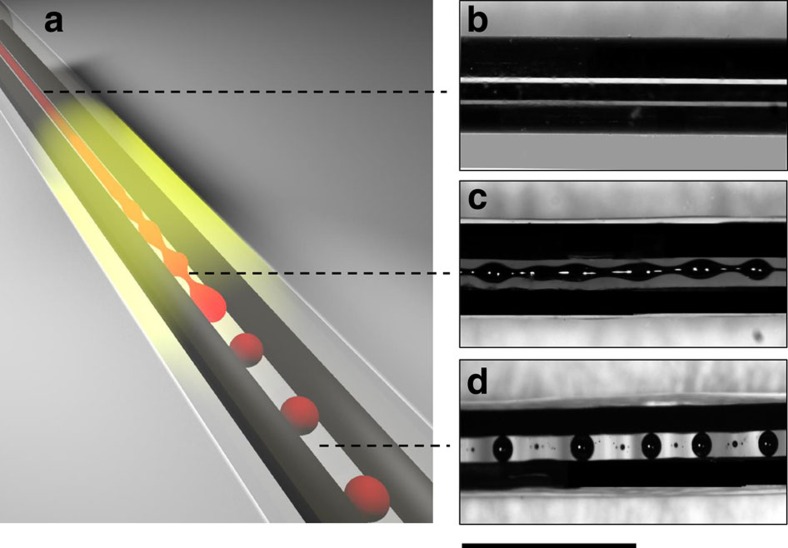
Evolution of selective break-up in fibres. (**a**) Illustration of the selective break-up process in the fibre. (**b**) Optical micrograph of a fibre with a semiconducting core radius of 50 μm designed to undergo selective break-up—before break-up. (**c**) Optical micrograph of a fibre during the onset of break-up process—the inner core develops instability, while the electrodes are kept continuous. (**d**) Optical micrograph of a fibre after break-up with chalcogenide glass spheres with a radius of 107 μm, connected to continuous electrodes. (Scale bar, 1 mm).

After the draw, the fibres were heated isothermally on a hot plate at 230 °C, a temperature at which both the semiconducting core and the polymeric cladding are in liquid / soft state with a low enough viscosity such that the break-up happens reasonably fast. This heating was conducted until the desired structure was achieved as illustrated in Fig. 3a. Figure 3b–d show the evolution of the fibre structure during the break-up of a 50 μm radius central core fibre. The chalcogenide core breaks into spheres with periodic pitch, while the electrodes remain intact (see Supplementary Movie 1). A self-assembled fibre structure is achieved with a broken translational symmetry along the fibre axis. This approach is universal, scalable in size and can be used over a wide set of materials, paving the way for a new set of hitherto unachievable fibre devices. The sphere size is relatively uniform, but should be considered polydisperse, with a standard deviation to mean ratio of 9%. The sphere radius and subsequent pitch period distribution could be attributed to distribution of initial perturbation amplitudes, which would result in a dispersion in sphere radii. Similar results were obtained for spheres with radii of 5 and 11 μm (see Supplementary Note 2, Supplementary Figs 2–4 and Supplementary Table 2).

The ratio between the sphere radius, *R,* and the core radius, *a*, can be derived from volume conservation as 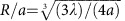, where *λ* is the period spacing between the spheres along the fibre axis. Along with the Rayleigh condition—*λ*≥2*πa* (refs 11, 12), the resulting sphere radius is larger than the initial core radius. As the capillary break-up starts from an arbitrary perturbation in the core radius, it is expected that the distance between two adjacent spheres should correspond to the perturbation wavelength with the minimal time constant (or the maximal growth rate). The perturbation wavelength that is associated with the shortest growth time constant in our case, according to equation (1) is 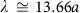. Assuming volume conservation, we get that *R*=2.17*a*. These relations, which are derived by the Tomotika model^8^, were found to be in a good agreement with our experimental results obtained for different core radii (See Supplementary Note 1, Supplementary Fig. 5, Supplementary Table 2). We note that the distance between the electrodes and the semiconducting core in the preform was designed such that it is approximately equal to (*R*−*a*)*β*, where *β* is the draw down ratio.

### Photoresponse characterization

**Figure 4:**
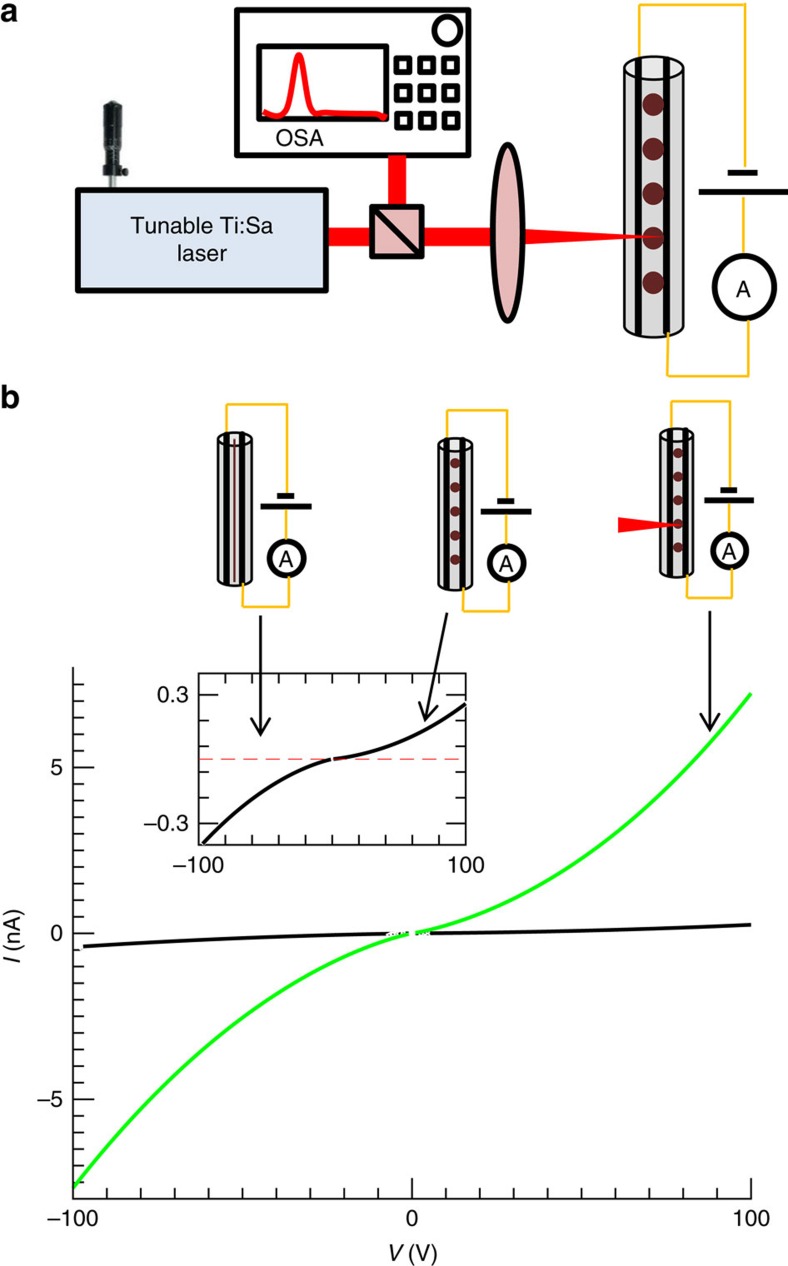
Photodetection measurement set-up and photoresistance characterization. (**a**) Schematic representation of the experimental set-up used to characterize photodetection and resonative photoresponse of selectively broken fibres. The fibres are illuminated with a Ti:Sapphire laser (operated at a wavelength of 760±1 nm, and power of 55 mW), while the laser power, spectrum and fibre photoresponse is being recorded simultaneously. (**b**) Main figure—measured current versus voltage curve of the photodetecting fibre with spheres with an average radius of 11 μm under laser illumination (green), compared with the dark current (black). The set-up shown in **a** was used, while illuminating a single sphere; Inset—IV curve obtained in the dark (black), the dark current of the fibres prior to break-up (dashed red line)—shows no measurable current due to lack of contact between the semiconducting core and the electrodes.

The electro-optical behavior of the fibres post break-up was characterized by illuminating the fibre structure with a continuous wave Ti:Sapphire laser source with a wavelength of 760±1 nm, power of 55 mW, through a spherical lens that resulted in spot full width half maximum (FWHM) radius of 22 μm. The light was incident with an angle of 90° with regards to the fibre axis, as shown in Fig. 4a. The electric current was recorded for various forward and reverse voltages, under dark and with laser illumination conditions.

Figure 4b shows the IV curve of a fibre that contained spheres with a radius of 11 μm. The current at a given voltage is considerably higher when it is illuminated—exhibiting a noticeable photo resistive effect. The theoretical dependence of the current on the applied voltage is described in Supplementary Note 3, while further experimental results made with 5 μm spheres are shown in Supplementary Fig. 6.

The self-assembled structure has photodetecting properties, and it is the first photodetecting structure obtained through selective break-up in fibres. This result demonstrates the ability to integrate electronic devices in two steps—fibre draw and subsequent heat treatment. The photoresistive behavior of these fibres is similar, in principle, to previously demonstrated fibres^13^,^14^,^15^ that contained continuous semiconducting core and conductors in contact, although a different semiconducting material was used. The measured dark and photo currents in the spherical semiconducting fibres depend on the geometrical dimensions of the devices, such as the small contact area between the electrodes and the core, which influence the absolute value of both currents, together with the excitation wavelength and the laser power (as fully described in Supplementary Note 3 and Supplementary Tables 3–6).

### Resonative photoresponse characterization

Spherically shaped structures are perfect candidates for resonant light coupling, and are extensively used as optical filters or gain media^16^,^17^. Chalcogenide glass resonators were fabricated previously using a multitude of approaches^18^,^19^,^20^,^21^. The majority characterization attempts of the resulting devices have focused on the optical properties of a single sphere, manipulated individually and evanescently coupled to a tapered fibre or a waveguide. Using the approach mentioned above, we were able to obtain a large amount of in-fibre integrated chalcogenide spheres contacted by continuous electrodes in parallel. Optical resonances in this configuration can be directly coupled to photodetection, which results in increased photoconduction, that is directly registered by electronics interfaced to the device electrodes. This modality was not previously achievable with photodetecting fibres^13^,^14^,^15^ as the core shape was cylindrical, whereas, with spherical semiconducting core, photoelectric resonances become available.

**Figure 5:**
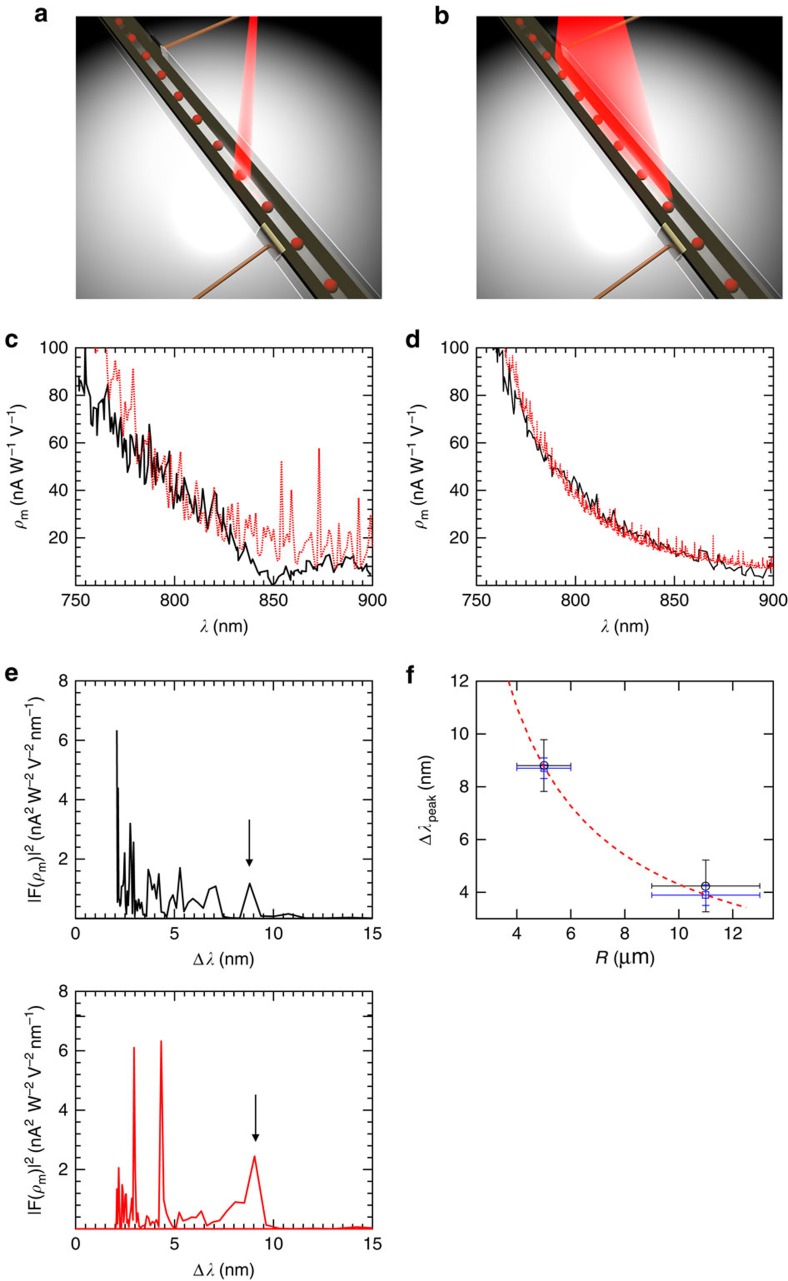
Resonative photodetection characterization. (**a**) Illustration of the fibre characterization area where only one sphere is excited by the laser (through a spherical lens). The laser wavelength was tuned continuously. The measurements of the current, wavelength and the laser power were recorded, as shown in Fig. 4a. (**b**) Illustration of the fibre characterization area where several spheres are excited by the laser (through a cylindrical lens). (**c**) Experimentally measured responsivity (black) compared with theoretically calculated responsivity (red) as a function of wavelength for a fibre with a sphere radius of 5 μm. The fibre was illuminated by a spherical lens in a single sphere excitation as described in **a**. (**d**) Experimentally measured responsivity (black) compared with theoretically calculated responsivity (dashed red) as a function of wavelength for a fibre with a sphere radius of 5 μm. The fibre was illuminated by a cylindrical lens in a multiple sphere excitation as described in **b**. Here the polydispersity of the spheres was taken into account (see Supplementary Note 4). The resonant peaks are less pronounced due to polydispersity of the spheres. (**e**) The FFT density of the experimental responsivity (black) and the theoretical responsivity (red) as a function of wavelength between two adjacent resonant peaks are described in the upper and the lower graphs, respectively, calculated for a single sphere excitation. The location of the first order peak in the experimental and the theoretical results is Δ*λ*_peak_=8.5±0.5 nm and Δ*λ*_peak_=8.8 nm, respectively (marked by an arrow). (**f**) Comparison between the theoretical peak location of the FFT density as a function of sphere radius (red dashed-line), for the case of single sphere and the experimental results for single- (black circle) and multiple-sphere excitation (blue square) is presented. Error bars correspond to 95% confidence interval, both for the wavelength peak location and the sphere radii.

Such photoelectric resonances in a single sphere were recently reported^22^. Mie's theory^23^,^24^ is used here to explain the resulting resonant behavior of our fibre devices. The resonances were characterized using an experimental set-up illustrated in Fig. 4a. The fibre was illuminated with a continuous wave radiation of a Ti:Sapphire laser with a spectral width (FWHM) below 1 nm. This laser source was chosen since it delivers up to 100 mW narrow band radiation at the desired wavelength range of 750 to 900 nm—near the absorption edge of As_2_Se_5_. Two different modes of illumination were investigated: a single sphere excitation—by focusing the light through a spherical lens as shown in Fig. 5a (FWHM of 22 μm), and a multi-sphere excitation—by illumination through a cylindrical lens, as shown in Fig. 5b (two-dimensional Gaussian beam with a FWHM of 462 × 180 μm), to produce an elliptical light spot. For a given sphere radius, the photocurrent was mapped as a function of wavelength while the laser wavelength and the power were concurrently monitored. The photoelectric responsivity, *ρ*_m_, (as defined in Supplementary Note 3, Supplementary Equation 17), was measured as a function of wavelength, and is shown in Fig. 5c for a 5 μm sphere radius in the case of single-sphere excitation, while Fig. 5d shows results for multi-sphere excitation. The theoretical prediction based on Mie theory, both for single and multi-sphere excitation was calculated and is expressed in terms of the calculated responsivity, which was shown to be proportional to the normalized absorption cross section −*Q*_abs_. (See Supplementary Notes 3,4, Supplementary Figs 7 and 8).

The results in Fig. 5c,d suggest that both the measured and the theoretically calculated responsivity contain resonant, periodic features. Due to sphere polydispersity, Mie resonances are less pronounced in the case of multiple sphere excitation, consistent with previous findings^25^,^26^. The polydispersity was taken into account (see Supplementary Note 4) when estimating the theoretical responsivity for multi-sphere excitation. This was performed by randomly drawing a finite number (equal to the number of illuminated spheres) of sphere radii out of a normal distribution with the measured radius mean and corresponding s.d. The responsivity was calculated for every sphere, and then the total responsivity was computed by averaging the individual contributions of each sphere, which is shown by the red curve in Fig. 5d.

Mie resonances are expected to be periodic in the k-space (or the frequency domain)^24^,^27^, thus to quantitatively characterize the periodic features recorded in the measurements and obtained in the calculations, we performed a fast Fourier transform (FFT) analysis. We first transform the measurements in to an optical frequency domain (or k-space), we then obtain the FFT and finally rescale the horizontal axis to wavelength units to highlight the Mie scattering free spectral range (FSR). Figure 5e describes the FFT analysis of the measured responsivity and the calculated theoretical responsivity for a single 5 μm radius sphere excitation (analysis for other sphere radii and multiple sphere excitation regime is shown in Supplementary Figs 9–11). Since the sphere size is much larger than the wavelength used for characterization, multiple resonant modes are allowed to exist. Thus, we have focused our efforts on finding the primary mode which has the largest FSR. A resonance frequency at 8.5±0.5 nm is present for the measured responsivity of single sphere excitation and is 8.8 nm for the calculated responsivity (marked by arrows), supporting the claim that resonances were present in the responsivity of the photodetectors due to the spherical shape of the detecting elements. This FFT peak is broader for multi-sphere excitation due to the polydispersity of the spheres (Supplementary Fig. 11).

The experimental and theoretical dependence of the resonant peak on the sphere size and excitation regime is summarized in Fig. 5f. A good agreement is achieved between theory and experiment. In particular, the experimental results confirm the theoretically derived inverse proportionality dependence of the FSR on the sphere radius (see Supplementary Note 4). It is also shown that both single sphere and multi-sphere excitations showed very similar FSRs, supporting the prediction that the resonances are due to intra-sphere light interferences (which are dominant in our case) as opposed to inter-sphere interactions. Intra-sphere interactions are most likely dominant due to the regime of excitation—perpendicular to the fibre axis.

## Discussion

The results mentioned above, demonstrate in-fibre self-assembly of discrete semiconducting micro-spheres with axially continuous electrodes that form electrical contacts and produce fully packaged devices along meters of fibre. A fibre containing a semiconducting core and two conductive domains that are electrically insulated from each other. Subjected to heating, the Plateau–Rayleigh effect^11^,^12^ results in the formation of linear array of spheres having a larger radius than that of the initial semiconducting cylinder, establishing an electrical connection with the adjacent conductive buses. This approach provides a pathway to avoid semiconducting material contamination during prolonged contact at high temperature between conductors and semiconductors at the fibre draw step^13^,^14^,^15^, enabling new fibre architectures and functionalities that were previously unattainable, all electrically connected and fully packaged. These results pave the way to numerous possible fibre based devices such as a fully packaged p-n or p-i-n photodetectors with a ‘spherical—molecules' configuration—internal to the fibre, addressable through the continuous buses. This approach demonstrated how to achieve novel fibre structures in which components are brought into contact post draw, through the aforementioned selective break-up process.

## Methods

### Chalcogenide rod fabrication

The chalcogenide material (As_2_Se_5_, AMTIR4, amorphous materials) was inserted into a silica ampule with an inner diameter of 4 mm. The ampule was evacuated in vacuum, sealed and placed in a rocking furnace. It was heated at 650 °C for 10 h and then extracted from the ampule.

### Fibre fabrication

The fibres were fabricated by a multistep thermal draw, out of a macroscopic preform. 75 μm thick PC (Lexan) films were rolled on a teflon lined mandrel, and then fused in vacuum at 190 °C for 15 min. Two pockets were milled in the PC and two CPE (carbon black loaded low density polyethylene) electrodes (7.5 × 3 × 90 mm) were inserted in these channels, followed by additional layers of PC which were added to increase the preform diameter to 36 mm. After a second fusing step (vacuum, 190 °C, 40 min), the inner mandrel was removed and the chalcogenide rod was inserted to the hollow core of the preform. The preform was thermally drawn at a temperature of 300 °C, while maintaining a low stress in the fibre during the draw. The structure was scaled down by a factor of 40.

To further reduce the size of the inner core, additional films of PC were wrapped around the fibre until a diameter of 20 mm was reached. After a fusing step, two channels were milled in the cladding until the first fibre electrodes were exposed, and additional, larger CPE electrodes were inserted. Final layers of PC were wrapped around the preform until the preform thickness was 30 mm, which was subsequently fused and drawn down by a factor of 10–20 to achieve fibre core radii of 5 and 2.5 μm.

### Fibre capillary break-up

The drawn fibres were placed on a hot plate (Cole Parmer, Stable Temp) and heated at a temperature of 230 °C and simultaneously observed under a stereoscope (Nikon SMZ745T) until the core was broken up into a series of spheres. The continuous electrodes were exposed from the fibre cladding and connected to copper wires with a silver paint.

### Electrooptical characterization

Nd:YAG pumped Ti:Sapphire laser (Coherent Mira900) was used to characterize the photodetecting properties. The laser power and wavelength was sampled by a power meter (Newport 2936-R) and an optical spectrum analyser (Ando AQ6317B). The laser was focused on the spheres with spherical or cylindrical lenses with focal length of 50 mm. The laser was operated in a continuous mode. The current and voltage were measured and supplied by picoammeter measuring set-up (Kiethley 6487/6517A). All the collected data was recorded by a Labview software, while the wavelength of the laser was tuned mechanically with an in-lab installed motor.

### Data availability

The data that support the findings of this study are available from the corresponding author upon request.

## Additional information

**How to cite this article:** Rein, M. *et al*. Self-assembled fibre optoelectronics with discrete translational symmetry. *Nat. Commun.* 7:12807 doi: 10.1038/ncomms12807 (2016).

## Supplementary Material

Supplementary Information Supplementary Figures 1-11, Supplementary Tables 1-6, Supplementary Notes 1-4 and Supplementary References

Supplementary Movie 1 Evolution of selective fluid instability in multimaterial fibres. The inner core made of an amorphous semiconductor breaks up into a periodic series of spheres while two conductive buses made of conducting polyethylene composite stay continuous, when the fiber is heated at 230°C. The resulting structure is a self-assembled fiber photodetecting fiber with spherical photodetectors. Initial cylindrical semiconducting core radius - 50 μm; The spheres look spheroidic due to optical aberration induced by the high index refraction cladding.
