# Supplementary material for: Self-assembled fibre optoelectronics with discrete translational symmetry
Source: Nat Commun. 2016 Oct 4;7:12807. doi: 10.1038/ncomms12807 (PMC5059452; doi:10.1038/ncomms12807)
Supplement: Supplementary Information — Supplementary Figures 1-11, Supplementary Tables 1-6, Supplementary Notes 1-4 and Supplementary References [file ncomms12807-s1.pdf]

Supplementary Information

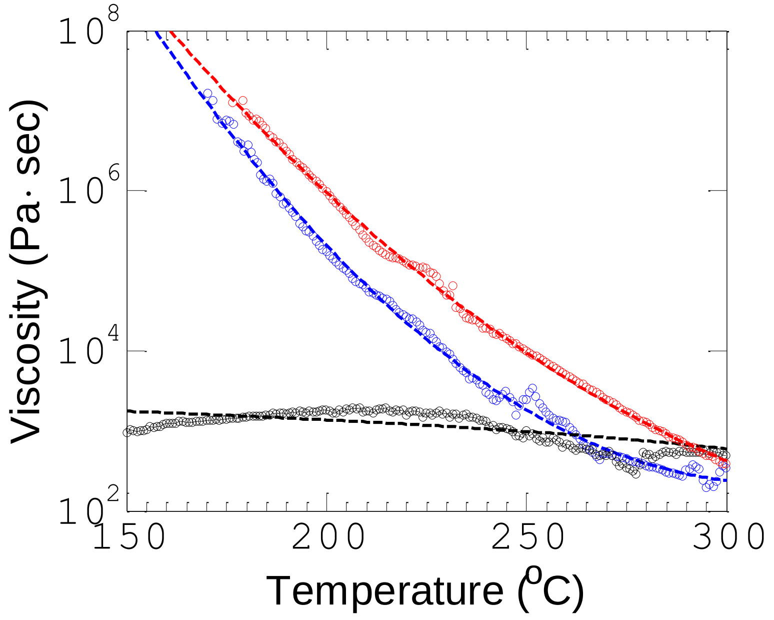

Supplementary Figure 1 – Viscosity of fibre components (PC cladding – blue; As_2Se_5 – red; CPE – black) as a function of temperature and fit to exponential functions (dashed lines). For more details, see Supplementary Note 1

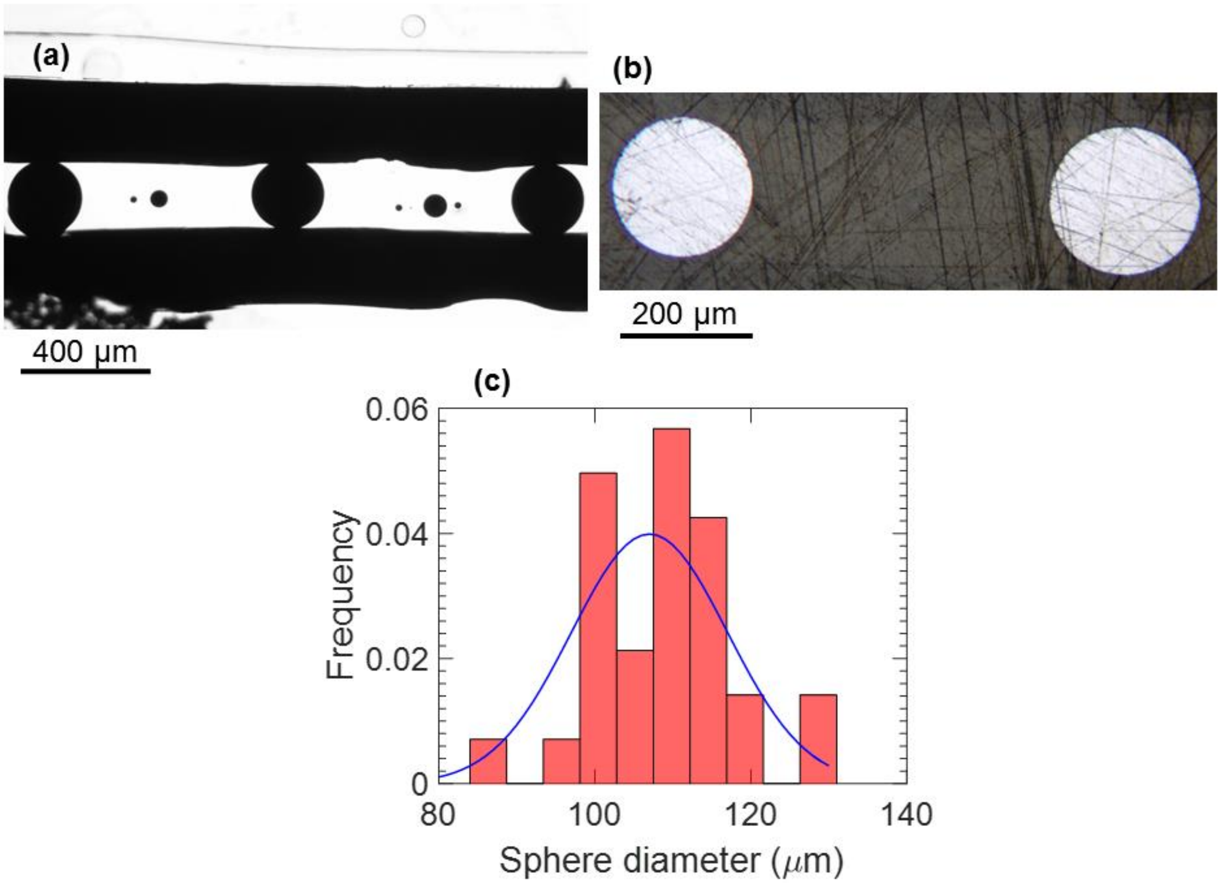

Supplementary Figure 2 – (a) Transmission optical micrograph of a fibre after break up, which contains semiconducting spheres with an average radius of 107µm, and CPE electrodes. (b) Reflection optical micrograph of the fibre shown in (a). (c) Sphere radius distribution estimation, from a sample of 30 spheres – average size - 107µm, standard deviation - 10µm. For more details, see Supplementary Note 2

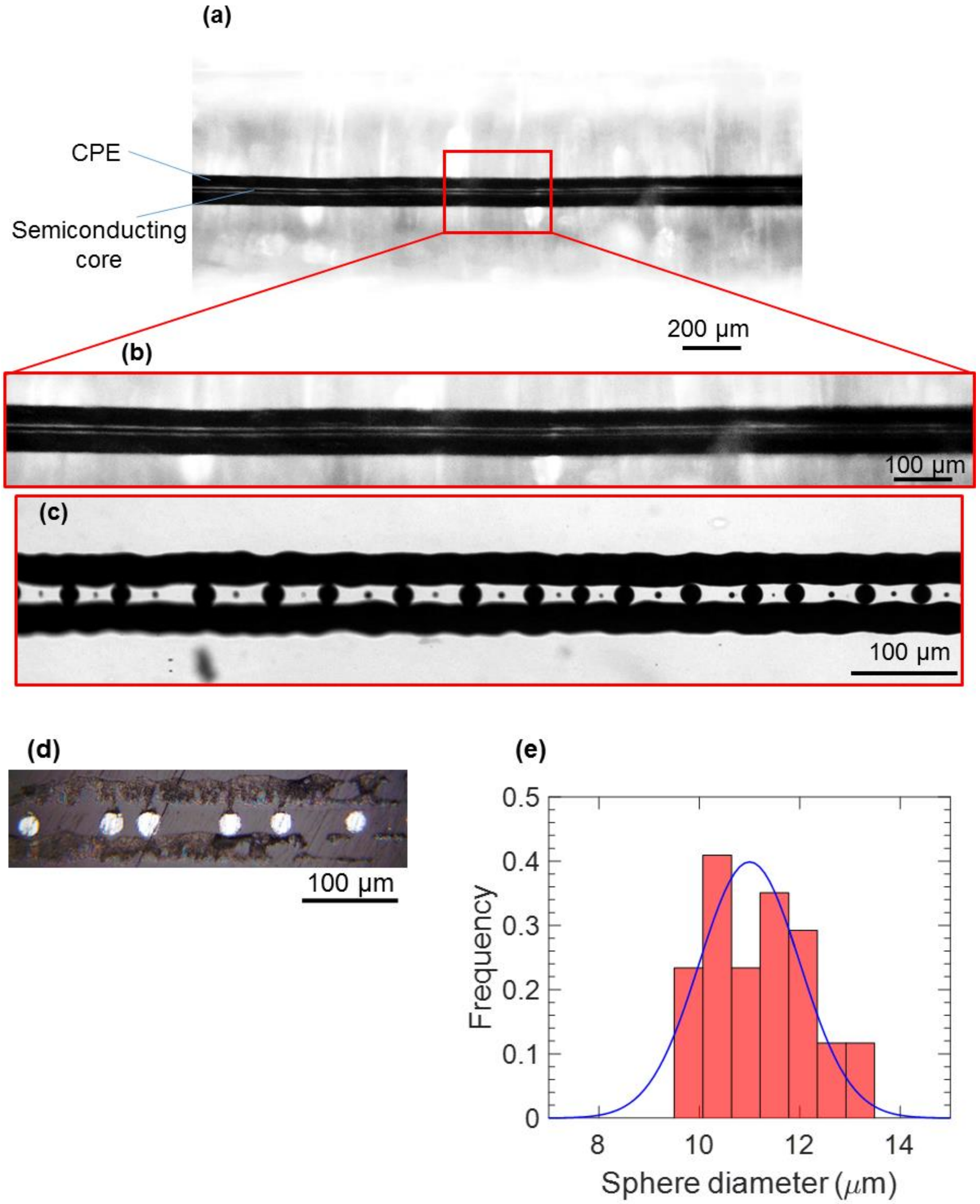

Supplementary Figure 3 – (a) Transmission optical micrograph of a fibre with a semiconducting core radius of 5µm designed to undergo selective break up – prior to break up. (b) Transmission optical micrograph of the active region in the fibre, shown by the red square in (a), shown with a greater magnification. (c) Transmission optical micrograph of a fibre after break up with chalcogenide glass spheres with an average radius of 11µm, connected to continuous electrodes. (d) Reflection optical micrograph of a fibre shown in (c). (e) Sphere radius distribution estimation, from a sample of 30 spheres – average size - 11µm, standard deviation - 1µm.

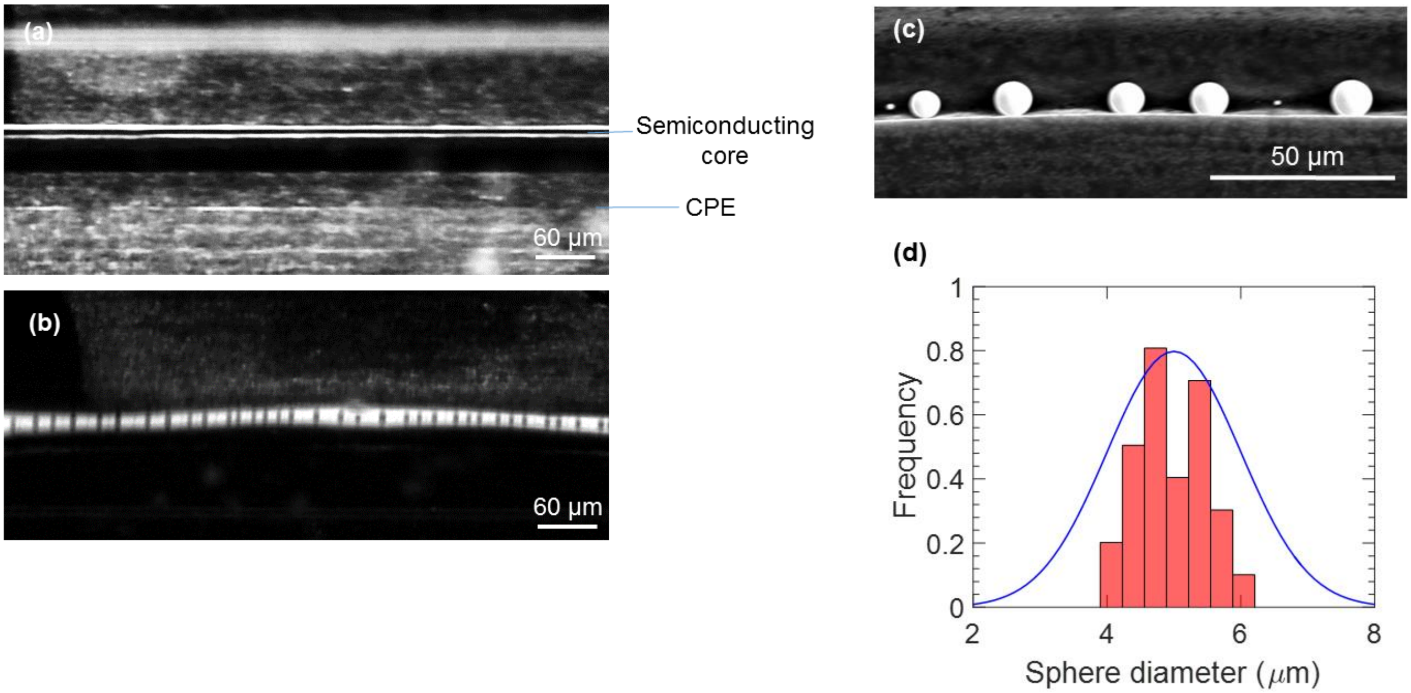

Supplementary Figure 4 – (a) Transmission optical micrograph of a fibre with a semiconducting core radius of 2.5µm designed to undergo selective break up – prior to break up. (b) Transmission optical micrograph of a fibre after break up with chalcogenide glass spheres with an average radius of 5µm, connected to continuous electrodes. (c) Scanning electron microscopy micrograph of a fibre with average sphere radius of 5µm, after chemical removal of the fibre cladding. (d) Sphere radius distribution estimation, from a sample of 30 spheres – average size - 5µm, standard deviation - 0.5µm.

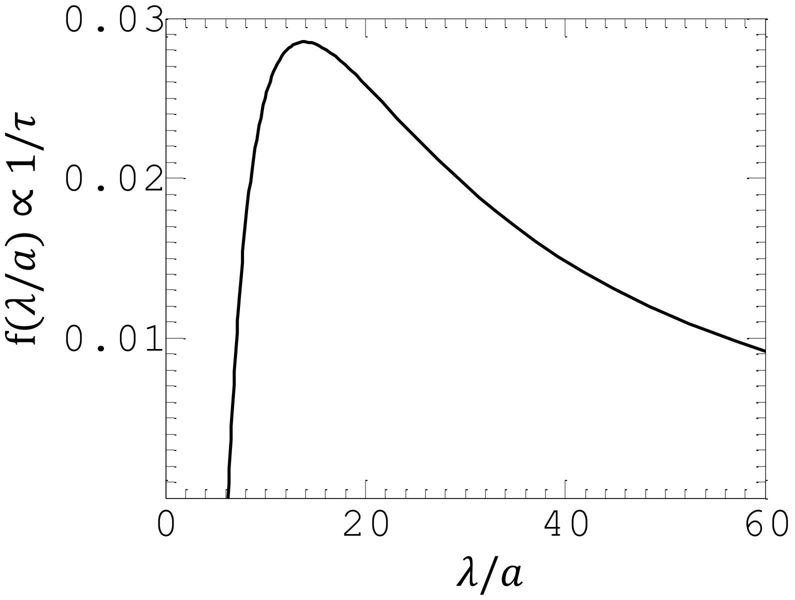

Supplementary Figure 5 – f – a function proportional to the perturbation growth rate ($1/\tau$) as a function of $\boldsymbol{\lambda/a}$ at 230°C. For more details, see Supplementary Note 1

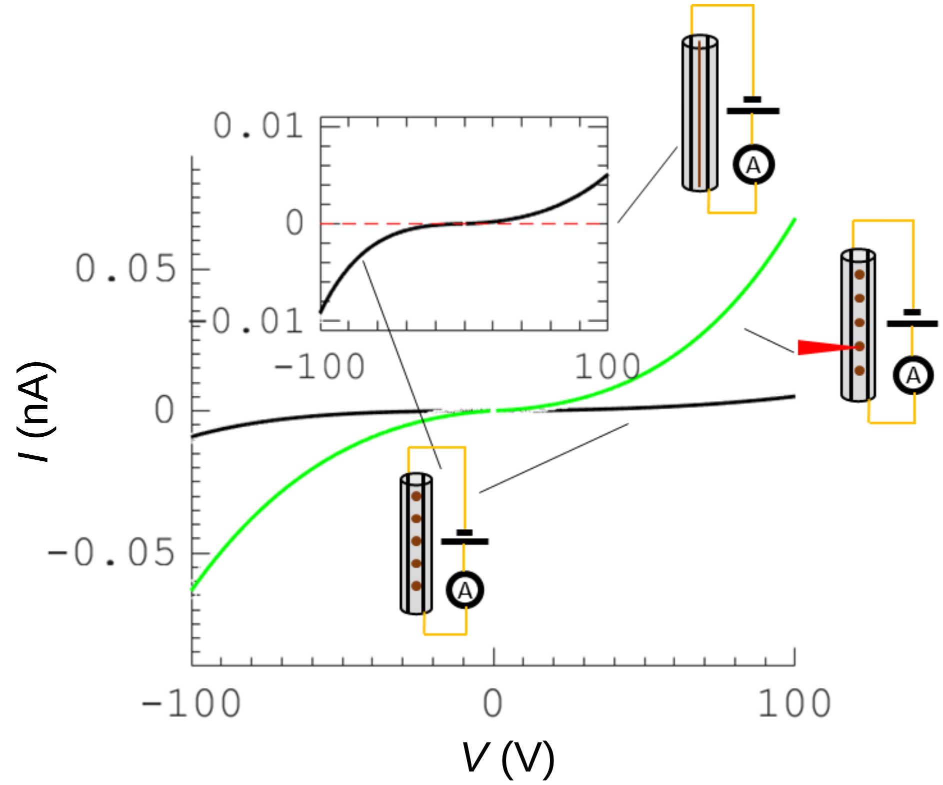

Supplementary Figure 6 - Main figure - Measured current versus voltage curve of the photodetecting fibre with spheres with an average radius of 5 μm under laser illumination (green), compared to the dark current (black). The setup shown in Figure 4(a) was used, while illuminating a single sphere, as shown in Figure 5(a); Inset - IV curve obtained in the dark (black), the dark current of the fibres prior to break up (red) – shows no measurable current due to lack of contact between the semiconducting core and the electrodes.

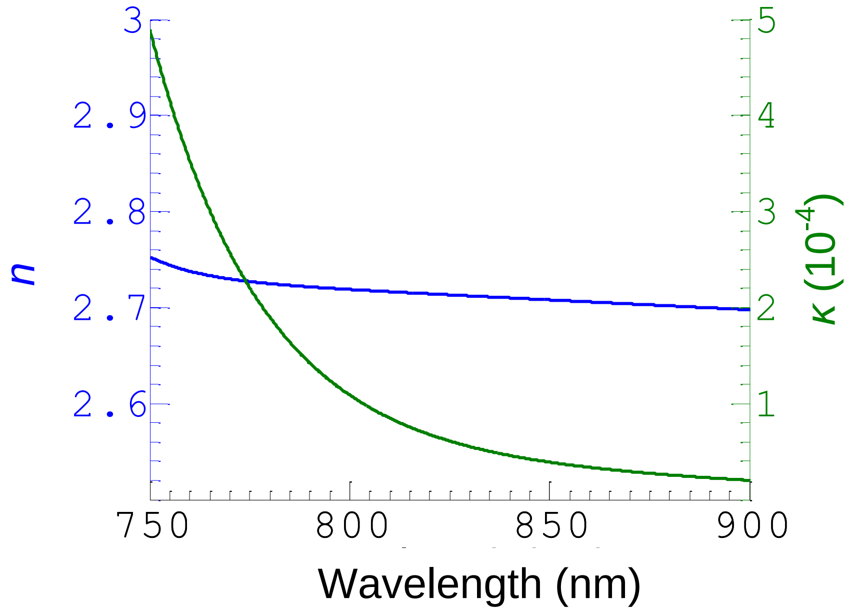

Supplementary Figure 7 – Index of refraction and extinction coefficient of As_2Se_5. For more details, see Supplementary Note 4

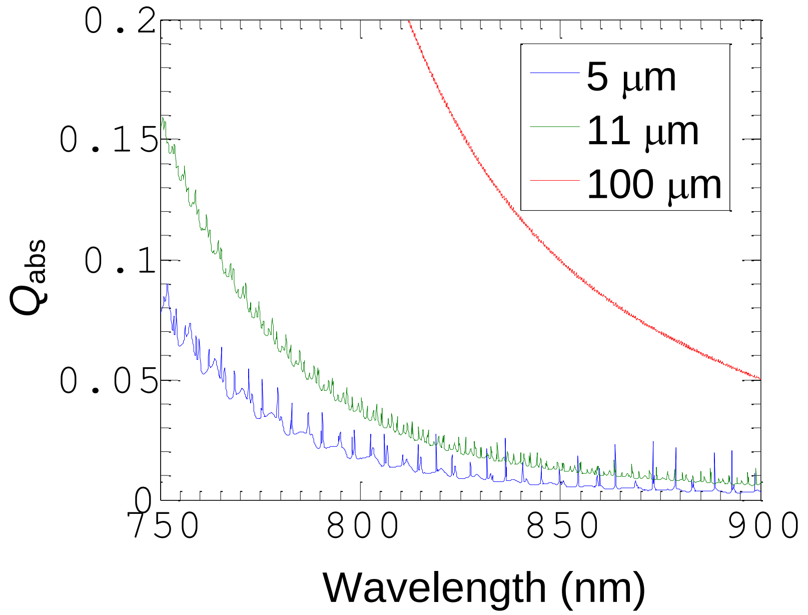

Supplementary Figure 8 – Q_{abs} calculated by Mie theory vs. wavelength for different sphere diameters. For more details, see Supplementary Note 4

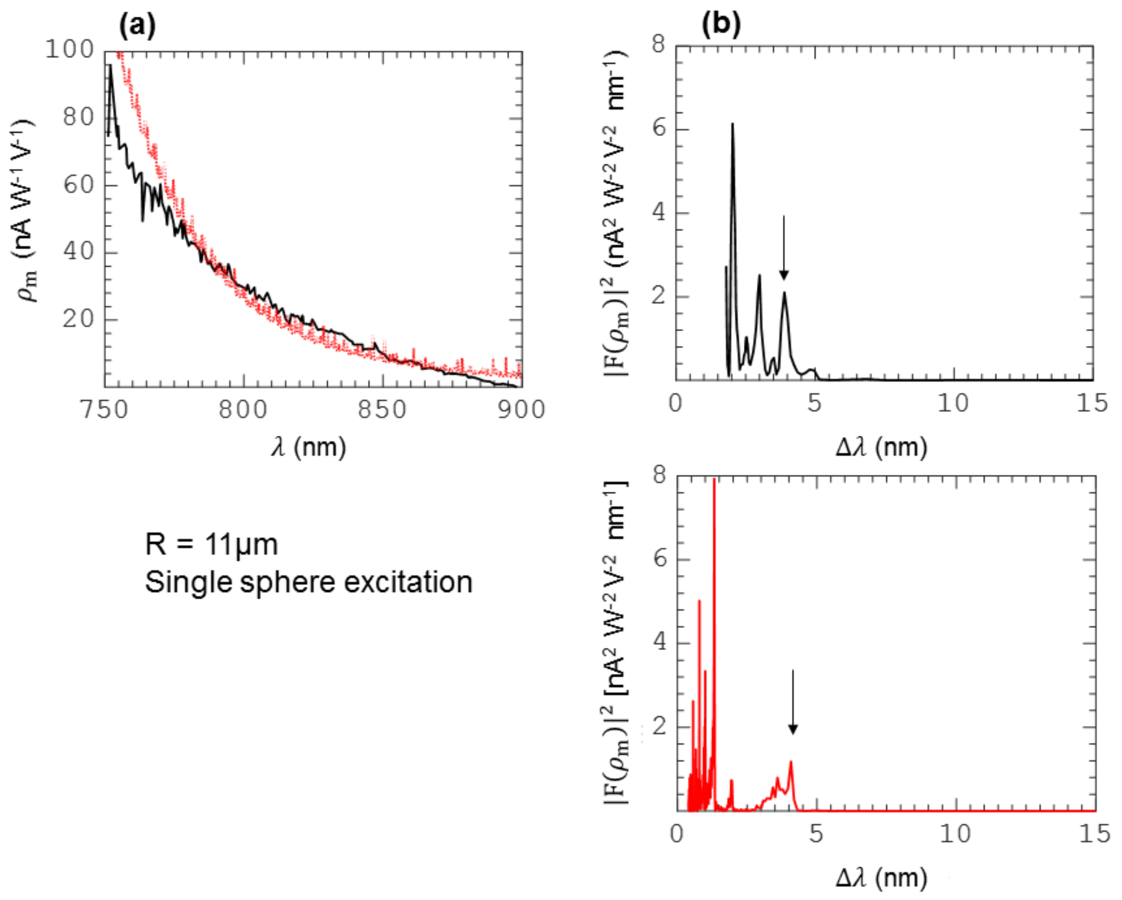

Supplementary Figure 9 - (a) Experimentally measured responsivity (black) compared with theoretically calculated responsivity (red), as a function of wavelength for a fibre with a spheres radius of 11 µm. The fibre was illuminated by a spherical lens in a single sphere excitation as described in Figure 5(a). (b) The FFT density of the experimental responsivity (black) and the theoretical responsivity (red) as a function of wavelength between two adjacent resonant peaks are described in the upper and the lower graphs, respectively. The location of the first order peak in the experimental and the theoretical results is $\Delta\lambda_{peak}$= 3.9±0.3 nm and $\Delta\lambda_{peak}$ = 4.0nm, respectively (marked by an arrow).

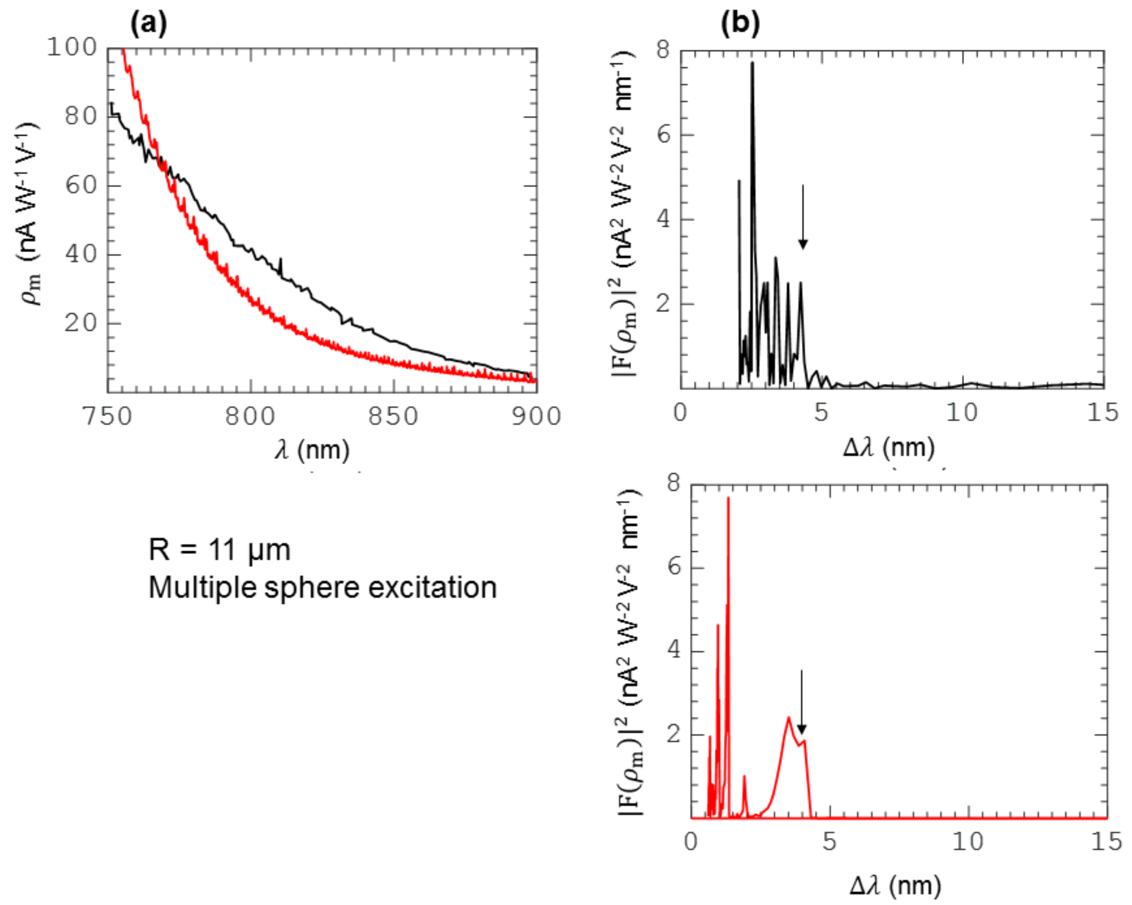

Supplementary Figure 10 - (a) Experimentally measured responsivity (black) compared with theoretically calculated responsivity (red), as a function of wavelength for a fibre with a spheres radius of 11 μm. The fibre was illuminated by a cylindrical lens in a multiple sphere excitation as described in Figure 5(b). (b) The FFT density of the experimental responsivity (black) and the theoretical responsivity (red) as a function of wavelength between two adjacent resonant peaks are described in the upper and the lower graphs, respectively. The location of the first order peak in the experimental and the theoretical results is $\Delta\lambda_{peak}$= 4.15±0.3 nm and $\Delta\lambda_{peak}$ = 4.2nm, respectively (marked by an arrow).

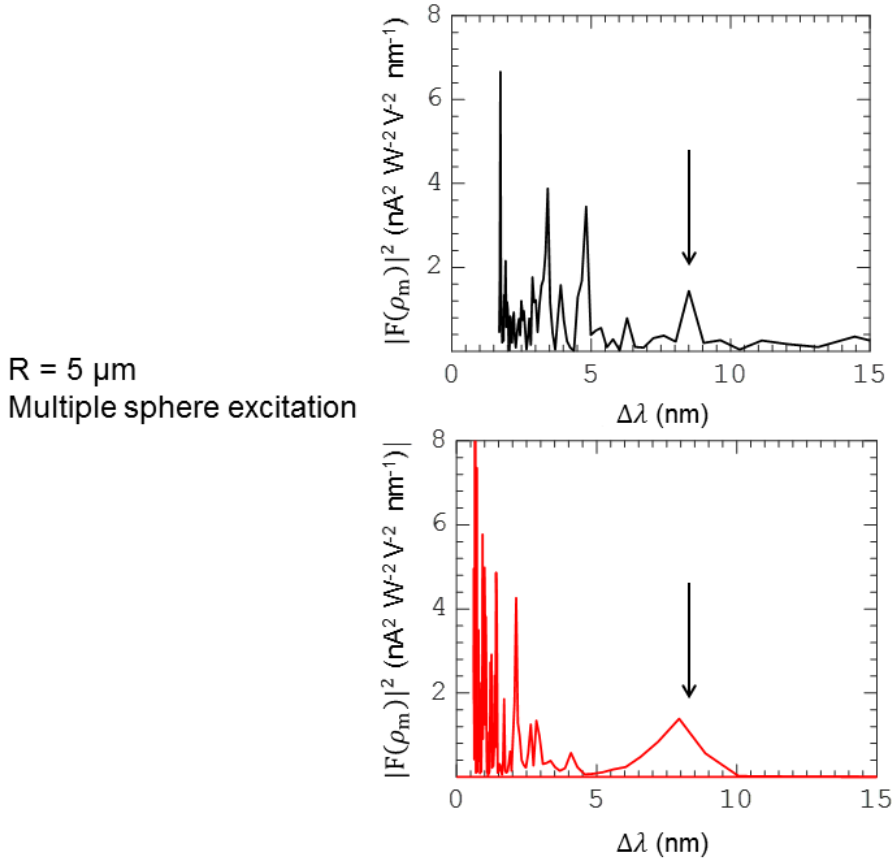

Supplementary Figure 11 - The FFT density of the experimental responsivity (black) and the theoretical responsivity (red) as a function of wavelength between two adjacent resonant peaks are described in the upper and the lower graphs, respectively, for fibres containing spheres with radius of 5 μm. Multiple excitation scheme. The location of the first order peak in the experimental and the theoretical results is marked by an arrow.

Property	Expression	As_2Se_5	CPE	PC
Cross sectional radius (μm)	a	50	72*	
Surface tension with PC[4,5] (mJ)	γ	114	30	
Viscosity at 230°C (Pa · s)	η	$7 \cdot 10^4$	$1.2 \cdot 10^3$	$2 \cdot 10^4$
Activation energy (eV)	E_a	2.02		2.31

*Effective cross sectional radius calculated from square cross section area.

Supplementary Table 1 – Fibre constituent properties. For more details, see Supplementary Note 1

Continuous core radius a (μm)	Sphere radius R (μm)	Period wavelength λ (μm)
50 ± 1	107 ± 10	660 ± 90
5 ± 0.1	11 ± 1	68 ± 12
2.5 ± 0.7	5 ± 0.5	35 ± 5

Supplementary Table 2 – Summary of the initial core and sphere radii and period wavelength of the sphere separation after the break up. Errors shown correspond to standard deviations. For more details, see Supplementary Note 1

Sphere radius R (µm)	A ($\mathrm{A\,V^{-1}}$)	B ($\mathrm{m^{0.5}\,V^{-0.5}}$)
5	$2 \cdot 10^{-15} \pm 1 \cdot 10^{-15}$	$9.2 \cdot 10^{-4} \pm 1.2 \cdot 10^{-4}$
11	$8 \cdot 10^{-13} \pm 4 \cdot 10^{-13}$	$6.9 \cdot 10^{-4} \pm 1.2 \cdot 10^{-4}$

Supplementary Table 3 – Summary of fit results. For more details, see Supplementary Note 3

Sphere Radius R (µm)	α_{geom}, single sphere	α_{geom}, multiple sphere
5	7.5	85.9
11	2	34.4

Supplementary Table 4 – Summary of geometric correction factors. For more details, see Supplementary Note 3

Property	Symbol	$R = 5\mu m$	$R = 11\mu m$
Carrier lifetime (s)	τ	$2 \cdot 10^{-5}$	
Voltage (V)	V	100	
Carrier mobility $(m^2 s^{-1})$	μ	$1.58 \cdot 10^{-9}$	
Transit time (s)	$\tau_{transit}$	$6.3 \cdot 10^{-4}$	$6.1 \cdot 10^{-3}$
Carrier collection efficiency	ζ	0.031	$3.25 \cdot 10^{-3}$
Dark current (A)	I_{dark}	$1.3 \cdot 10^{-11}$	$1.88 \cdot 10^{-10}$
Sphere periodicity wavelength (μm)	λ	68	35
Concentration of intrinsic carriers [10] (m^{-3})	n_{dark}	$1.3 \cdot 10^{17}$	
Specimen length (m)		0.05	

Supplementary Table 5 – Properties used for theoretical responsivity calculation. For more details, see Supplementary Note 3

Sphere Radius R (μm)	ρ_m Single Sphere $(nA\ W^{-1}\ V^{-1})$	ρ_m Multiple Sphere $(nA\ W^{-1}\ V^{-1})$	Calculated ρ_m $(nA\ W^{-1}\ V^{-1})$
5	19.3	12.5	20.5
11	14.4	14.1	19.1

Supplementary Table 6 – Summary of the measured normalized responsivity and the calculated responsivity from theory. For more details, see Supplementary Note 3

Supplementary Note 1: Timescale for break up and characteristic period

Tomotika model[1] predicts the perturbation growth rate of a Rayleigh-Plateau instability[2,3] in a "cylindrical thread of a viscous liquid surrounded by another viscous fluid":

$$(1) \qquad \tau = \tau_0/[(1-x^2)\Phi(x, \mu_{\mathrm{inner}}/\mu_{\mathrm{clad}})]$$

Where $x \equiv 2\pi a/\lambda$, a is the radius of the inner fluid, either the semiconducting core or the electrodes; $\tau_0 \equiv 2a\mu_{\mathrm{clad}}/\gamma$, μ_{clad} and μ_{inner} are the viscosity of the cladding and the inner fibre component, respectively; γ is the interfacial surface tension between the two liquids. τ and $1/\tau$ are the perturbation growth time and rate respectively, and λ is the periodicity of the spheres that are present in the fibre after break up. Function Φ has the following form[1]:

$$\Phi\left(x, \frac{\mu_{\mathrm{inner}}}{\mu_{\mathrm{clad}}}\right) = \frac{\mathrm{N}\left(x, \frac{\mu_{\mathrm{inner}}}{\mu_{\mathrm{clad}}}\right)}{\mathrm{D}\left(x, \frac{\mu_{\mathrm{inner}}}{\mu_{\mathrm{clad}}}\right)}$$

With

$$\mathrm{N}\left(x, \frac{\mu_{\mathrm{inner}}}{\mu_{\mathrm{clad}}}\right) \equiv \mathrm{I}_1(x)\Delta_1 - \{x\mathrm{I}_0(x) - \mathrm{I}_1(x)\}\Delta_2$$

$$\mathrm{D}\left(x, \frac{\mu_{\mathrm{inner}}}{\mu_{\mathrm{clad}}}\right) \equiv \left(\frac{\mu_{\mathrm{inner}}}{\mu_{\mathrm{clad}}}\right)\{x\mathrm{I}_0(x) - \mathrm{I}_1(x)\}\Delta_1 - \left(\frac{\mu_{\mathrm{inner}}}{\mu_{\mathrm{clad}}}\right)\{(x^2+1)\mathrm{I}_1(x) - x\mathrm{I}_0(x)\}\Delta_2$$
$$- \{x\mathrm{K}_0(x) + \mathrm{K}_1(x)\}\Delta_3 - \{(x^2+1)\mathrm{K}_1(x) + x\mathrm{K}_0(x)\}\Delta_4$$

In these expressions Δ_1, Δ_2, Δ_3 and Δ_4 are all functions of x expressed in determinantal form as follows:

$$\Delta_1 = \begin{vmatrix} x\mathrm{I}_0(x) - \mathrm{I}_1(x) & \mathrm{K}_1(x) & -x\mathrm{K}_0(x) - \mathrm{K}_1(x) \\ \mathrm{I}_0(x) + x\mathrm{I}_1(x) & -\mathrm{K}_0(x) & -\mathrm{K}_0(x) + x\mathrm{K}_1(x) \\ \frac{\mu_{\mathrm{inner}}}{\mu_{\mathrm{clad}}} x\mathrm{I}_0(x) & \mathrm{K}_1(x) & -x\mathrm{K}_0(x) \end{vmatrix}; \ \Delta_2 = \begin{vmatrix} \mathrm{I}_1(x) & \mathrm{K}_1(x) & -x\mathrm{K}_0(x) - \mathrm{K}_1(x) \\ \mathrm{I}_0(x) & -\mathrm{K}_0(x) & -\mathrm{K}_0(x) + x\mathrm{K}_1(x) \\ \frac{\mu_{\mathrm{inner}}}{\mu_{\mathrm{clad}}} \mathrm{I}_1(x) & \mathrm{K}_1(x) & -x\mathrm{K}_0(x) \end{vmatrix}$$

$$\Delta_3 = \begin{vmatrix} \mathrm{I}_1(x) & x\mathrm{I}_0(x) - \mathrm{I}_1(x) & -x\mathrm{K}_0(x) - \mathrm{K}_1(x) \\ \mathrm{I}_0(x) & \mathrm{I}_0(x) + x\mathrm{I}_1(x) & -\mathrm{K}_0(x) + x\mathrm{K}_1(x) \\ \frac{\mu_{\mathrm{inner}}}{\mu_{\mathrm{clad}}} \mathrm{I}_1(x) & \frac{\mu_{\mathrm{inner}}}{\mu_{\mathrm{clad}}} x\mathrm{I}_0(x) & -x\mathrm{K}_0(x) \end{vmatrix}; \ \Delta_4 = \begin{vmatrix} \mathrm{I}_1(x) & x\mathrm{I}_0(x) - \mathrm{I}_1(x) & \mathrm{K}_1(x) \\ \mathrm{I}_0(x) & \mathrm{I}_0(x) + x\mathrm{I}_1(x) & -\mathrm{K}_0(x) \\ \frac{\mu_{\mathrm{inner}}}{\mu_{\mathrm{clad}}} \mathrm{I}_1(x) & \frac{\mu_{\mathrm{inner}}}{\mu_{\mathrm{clad}}} xI_0(x) & \mathrm{K}_1(x) \end{vmatrix}$$

Where $\mathrm{I_n}$ and $\mathrm{K_n}$ are the modified Bessel functions of the first and second kind respectively.

As to our problem, we used Tomotika model to evaluate the timescales for break up, as a function of temperature, for the different components in our fibre structure. The cross sectional radius and the surface tension were assumed to be constant with regards to the temperature. The values of the cross sectional radius and the surface tension with PC that were used are summarized in Supplementary Table 1.

The viscosity of the fibre constituents were measured under varying temperature by rheometry, and fit to exponential functions, as the viscosity depends exponentially on the temperature. These results are shown in Supplementary Figure 1.

The viscosity of PC and As_2Se_5 has been fitted to Arrhenius function: $\eta = \eta_0 \exp(E_\mathrm{a}/kT)$, where E_a is often referred to as the activation energy, such that

$\eta_0 = 1.29 \cdot 10^{-19} \pm 4.9 \cdot 10^{-20}\ \mathrm{Pa \cdot s}$ and $E_\mathrm{a} = 2.31 \pm 0.02\ \mathrm{eV}$ for PC;

$\eta_0 = 3.9 \cdot 10^{-16} \pm 5 \cdot 10^{-17}\ \mathrm{Pa \cdot s}$ and $E_\mathrm{a} = 2.02 \pm 0.01\ \mathrm{eV}$ for As_2Se_5. The viscosity of CPE weakly depends on the temperature, and was taken as a constant.

Based on Tomotika model (Supplementary Equation 1), we calculate the characteristic rate for break up ($1/\tau$) for every component in the fibre. The characteristic rate corresponds to the maximal perturbation growth rate possible, for a given set of parameters. This maximal rate (or minimal τ) is obtained for a characteristic wavelength – λ, which is frequently referred to as Tomotika wavelength. This characteristic wavelength, λ, is therefore set by the maximum of the function $\mathrm{f}(\lambda/a) = [1-(2\pi a/\lambda)^2]\Phi(2\pi a/\lambda, \mu_\mathrm{core}/\mu_\mathrm{clad}) \propto 1/\tau$. The function f is shown in Supplementary Figure 5, as it was calculated for the semiconducting core of the fibre at the break up temperature of 230 $^\circ$C. The maximum in the function $\mathrm{f}(\lambda/a)$ is at $\lambda = 13.66a$, where a is the radius of the initial core. Similarly, we have calculated the values of τ, both for the electrodes and

the core, as a function of temperature and these are summarized in Figure 1(c) in the main text. At a given temperature, whenever the fibre is heated for a time that exceeds this timescale ($t > \tau$), we expect this component to break up.

Additionally, we have measured the radii of the initial semiconducting core and the radius of the resulting spheres and their period for three different initial core diameters. The results are summarized in Supplementary Table 2 and are in agreement with Tomotika[1] model.

Supplementary Note 2 – Additional Results and Data – Sphere Size and Shape

Supplementary figures 2-4 show the fibre structure for different fibre core sizes, which result in spheres radius of $107 \pm 10\mu\text{m}$, $11 \pm 1\mu\text{m}$ and $5 \pm 0.5\mu\text{m}$ respectively.

We have found that a normal distribution fits well the distribution of sphere radii, where the stated error is the standard deviation of the distribution. As can be seen in the Supplementary Figures 2-4, there are contacts between the vast majority of the spheres and the electrodes for spheres of radius of $107\mu\text{m}$ and $11\mu\text{m}$. Some of the $5\mu\text{m}$ spheres are not connected to the electrodes (Supplementary Figure 4), due to a shape fluctuation of the electrodes during the break up process. This is consistent with the relatively lower responsivity when measuring multiple spheres as described in Supplementary Note 3 (Supplementary Table 6, multiple sphere excitation).

Additionally, as can be seen in Supplementary Figures 2(b), 3(d) and 4(c), the semiconducting particles are very close to spherical. By measuring their diameter in two perpendicular directions allows us to estimate the ellipticity of these particles. The ratio between the minor and major axes yields in a maximum ratio of $\frac{\mathrm{d}_{\text{minor}}}{d_{\text{major}}} = 0.95$. Thus, we assume that the particles are very close to spherical.

Supplementary Note 3: Electrical behavior model of spherical semiconductor fibres

Our goal is to develop a model for the electric behavior of fibres that contain continuous electrodes and semiconducting spheres. We will do it in several steps.

1. Resistance of a single sphere: Since a resistance of a perfect sphere is infinite due to two singular contact points, we approximate this problem, by introducing a truncated sphere. Assuming the sphere has a homogeneous conductivity σ, an infinitesimal unit of the sphere has a resistance of: $\mathrm{d}\mathcal{R} = \mathrm{d}z/\sigma A(z)$, where z is the axis pointed between the two truncated caps, and $A(z)$ is the area that is defined by z, which is normal to the area of the sphere. Integrating $\mathrm{d}\mathcal{R}$ with respect to z gives the resistance of a sphere - $\mathcal{R}$:

$$(2) \quad \mathcal{R} = \frac{1}{\pi R\sigma}\ln\left(\frac{R+b}{R-b}\right) = \frac{1}{\pi R\sigma}\ln\left(\frac{1+\Omega}{1-\Omega}\right)$$

   Where R is the sphere radius, σ is the conductivity of the semiconductor, b is the distance between the truncated cap and the center of the sphere, and $\Omega = b/R$ is the contact factor, which is a function of the contact between the electrodes and the sphere.

2. The next step is to consider a fibre system which contains only one sphere that is connected to continuous electrodes. Here we assume that conductors have much smaller resistance than the semiconducting sphere, as it is in our case, and that there is no junction resistance between the electrodes and the sphere, at the contact.

   The carrier generation rate[6] is given by Supplementary Equation 3.

$$(3) \qquad \frac{(n - n_{\mathrm{dark}})}{\tau} = \frac{\eta\Phi}{\mathrm{Volume}} = \frac{3\eta\Phi}{4\pi R^3}$$

   Where n is the carrier density in the semiconductor, n_{dark} is the carrier density in the semiconductor without illumination, τ is the carrier lifetime, Φ is the photon flux and η is

the quantum efficiency. For the case of As_2Se_5 which is an amorphous arsenic selenium semiconductor, the electric transport is governed by holes[7].

Quantum efficiency of photo-generation and collection η for a spherical particle is given by Supplementary Equation 4:

$$(4) \quad \eta = \zeta \cdot Q_{\mathrm{abs}}$$

where $\zeta = \frac{\tau}{\tau + \tau_{\mathrm{transit}}}$ is the carrier collection efficiency that depends on the carrier lifetime and the transit time of the carrier from the location of formation to the collecting electrode; Q_{abs} is the normalized absorption cross section of a sphere, which is derived by Mie theory[8] as described in Supplementary Note 4. This value describes the interaction of electromagnetic radiation with the sphere and is the ratio between the absorbed photo-energy versus the incident energy, when the sphere is illuminated. Substituting Supplementary Equation 4 into Supplementary Equation 3 we can obtain the conductivity of a semiconducting sphere σ, which is given by Supplementary Equation 5:

$$(5) \quad \sigma = en\mu = \frac{3e\mu\zeta Q_{\mathrm{abs}}\Phi\tau}{4\pi R^3} + e\mu n_{\mathrm{dark}}$$

Where e is the electron charge and μ is the hole mobility.

The illuminated sphere resistance is given by substituting Supplementary equation 5 into Supplementary Equation 2:

$$(6) \quad \mathcal{R} = \frac{1}{\pi R\sigma} \ln\left(\frac{1+\Omega}{1-\Omega}\right) = \frac{4R^2 \ln\left(\frac{1+\Omega}{1-\Omega}\right)}{e\mu(4R^3\pi n_{\mathrm{dark}} + 3\zeta\tau\Phi Q_{\mathrm{abs}})}$$

3. In this step we look at a fibre that contains continuous electrodes, and a series of spheres all connected to these electrodes. At this step only one sphere is being illuminated while other are in the dark. In this case only the dark current should change. We now have $N = L/\lambda$ spheres in the system, where L is the length of the active fibre region that is defined by the distance

between the connections to the fibre electrodes by the external measurement setup and λ is the wavelength of periodicity which evolves during break up. Conductivity of the illuminated sphere is given by Supplementary equation 5, while the conductivity of the spheres that are not illuminated is:

$$(7) \quad \sigma_{\mathrm{dark}} = e\mu n_{\mathrm{dark}}$$

The resistance of the illuminated sphere is given by Supplementary equation 6 and the resistance of the spheres in the dark is:

$$(8) \quad \mathcal{R}_{\mathrm{dark}} = \frac{\ln\left(\frac{1+\Omega}{1-\Omega}\right)}{Re\pi\mu n_{\mathrm{dark}}}$$

Since these spheres are all connected in parallel, the total resistance $\mathcal{R}_{\mathrm{tot}}$ of the system is:

$$(9) \quad \frac{1}{\mathcal{R}_{\mathrm{tot}}} = \frac{1}{\mathcal{R}} + \frac{\frac{L}{\lambda} - 1}{\mathcal{R}_{\mathrm{dark}}} = \frac{e\mu(4R^3 L\pi n_{\mathrm{dark}} + 3\zeta\tau\Phi Q_{\mathrm{abs}}\lambda)}{4R^2 \ln\left(\frac{1+\Omega}{1-\Omega}\right)\lambda}$$

The current in the system I is thus:

$$(10) \quad I = \frac{V}{\mathcal{R}_{\mathrm{tot}}} = \frac{Ve\mu(4R^3 L\pi n_{\mathrm{dark}} + 3\zeta\tau\Phi Q_{\mathrm{abs}}\lambda)}{4R^2 \ln\left(\frac{1+\Omega}{1-\Omega}\right)\lambda}$$

Where V is the applied voltage.

The dark current which is present when the fibre is not illuminated ($\Phi = 0$) is given by Supplementary Equation 11.

$$(11) \quad I_{\mathrm{dark}} = \frac{ReL\pi\mu n_{\mathrm{dark}}}{\ln\left(\frac{1+\Omega}{1-\Omega}\right)\lambda} V$$

As can be seen in Figure 4(b) in the main text, the dark current is not linear with V. The reason for this nonlinear behavior is the dependence of the mobility of holes on the field, which is described by the Poole Frenkel effect[7,9]. The dependence of the mobility on the field is exponential as shown

in Supplementary equation 12:

$$(12) \quad \mu = \left(\frac{N_\mathrm{V}}{N_\mathrm{t}}\right)\mu_0 \exp\left(-\frac{U_0}{kT}\right)\exp\left(\frac{\beta E^{\frac{1}{2}}}{kT}\right)$$

where N_V is the density of states in the valence band, N_t is the density of the trapping centers, μ_0 is the carriers' microscopic mobility, U_0 is the thermal activation energy at zero field, and β is the Poole Frenkel constant. There are no exact values in the literature of As_2Se_5 for $N_\mathrm{V}, N_\mathrm{t}, \mu_0, U_0$ and β, but for a very similar and commonly used material – As_2Se_3, $\frac{N_\mathrm{V}}{N_\mathrm{t}}\mu_0 \approx 3 \cdot 10^{-3}\ \mathrm{m}^2\ V^{-1}\ \mathrm{s}^{-1}$, $U_0 = 0.43eV$ and $\beta = 1.85 \cdot 10^{-5} eV\ m^{0.5}\ V^{-0.5}$ as reported in[7].

The dark current has thus the following form:

$$(13) \quad I_\mathrm{dark} = \frac{ReL\pi V n_\mathrm{dark}}{\ln\left(\frac{1+\Omega}{1-\Omega}\right)\lambda}\left(\frac{N_\mathrm{V}}{N_\mathrm{t}}\right)\mu_0 \exp\left(-\frac{U_0}{kT}\right)\exp\left(\frac{\beta V^{\frac{1}{2}}}{kT(2R)^{\frac{1}{2}}}\right) = A \cdot V \exp\left(B\left(\frac{V}{2R}\right)^{\frac{1}{2}}\right)$$

where *A* and *B* are constants which were used as parameters to fit the expression in Supplementary Equation 13 to the experimental data. The fit results are summarized in Supplementary Table 3. According to the values of B in Supplementary Table 3 we find that the Poole Frenkel constant - β remains constant regardless of the sphere radius, ($\beta_{11\mu\mathrm{m}} = 17 \pm 3\ \mu\mathrm{eV\ m}^{0.5}\ \mathrm{V}^{-0.5}$; $\beta_{5\mu\mathrm{m}} = 23 \pm 3\ \mu\mathrm{eV\ m}^{0.5}\ \mathrm{V}^{-0.5}$; previously reported values[Error! Reference source not found.] of β - $18 \pm 2\ \mu\mathrm{eV\ m}^{0.5}\ \mathrm{V}^{-0.5}$), assuming that the temperature of the semiconductor didn't change during laser illumination.

On the other hand, the values of the pre-factor *A* change considerably between the experiments; this could not be explained just by the difference in sphere radius. These differences are most probably affected by the contact area between the sphere and the electrodes (mathematically described by Ω factor), which has a significant influence on the pre-factor *A*.

The responsivity ρ of the fibre, when only one sphere is illuminated is given by Supplementary equation 14:

$$(14) \qquad \rho = \frac{(I - I_{\mathrm{dark}})}{P} = \frac{3eV\zeta\lambda_{\mathrm{illum}}\mu\tau Q_{\mathrm{abs}}}{4hcR^2 \ln\left(\frac{1+\Omega}{1-\Omega}\right)}$$

where P is the power of the illumination that is incident on the sphere/s, and $\Phi = \frac{P\lambda_{\mathrm{illum}}}{hc}$. h is Planck's constant; c is the speed of light, λ_{illum} is the wavelength of the incident light.

This relation could further be simplified, by substituting Supplementary equation 11 into Supplementary equation 14:

$$(15) \quad \rho = \frac{3\zeta\lambda_{\mathrm{illum}}\tau Q_{\mathrm{abs}} I_{\mathrm{dark}}\lambda}{4hcR^3 L\pi n_{\mathrm{dark}}}$$

In order to compare the responsivities between various experiments, we have normalized it by the applied voltage:

$$(16) \qquad \rho_{\mathrm{m}} = \frac{\rho}{V} = \frac{3\zeta\lambda_{\mathrm{illum}}\tau Q_{\mathrm{abs}} I_{\mathrm{dark}}\lambda}{4hcR^3 L\pi n_{\mathrm{dark}} V}$$

This theoretical value was compared with the experimental values obtained for the different sphere sizes (radii of 5µm and 11µm) and for single and multiple sphere excitation.

The experimental normalized responsivity ($\rho_{\mathrm{m_{ex}}}$) was calculated by using Supplementary equation 17:

$$(17) \qquad \rho_{\mathrm{m_{ex}}} = \frac{I_{\mathrm{meas}} - I_{\mathrm{dark}}}{P_{\mathrm{meas}}}\alpha_{\mathrm{geom}}$$

Where I_{meas} is the measured current, P_{meas} is the total power supplied by the laser, and α_{geom} is a geometrical factor that takes into account the relative power that was incident on the spheres. Assuming that the laser beam flux is a two dimensional Gaussian, it is possible to calculate the

factor α_{geom} by the following expression:

$$(18) \quad I_{\mathrm{meas}} = \rho_{\mathrm{m}_{\mathrm{ex}}} P_{\mathrm{meas}} \int_S G(x,y)\mathrm{d}S$$

Where $G(x,y)$ is the Gaussian beam, experimentally measured, with FWHM s_{x}, s_{y} in the direction of the fibre axis and perpendicularly to the fibre axis, respectively. Comparing Supplementary equation 17 and Supplementary equation 18 gives us the value of α_{geom}:

$$(19) \quad \alpha_{\mathrm{geom}} = \frac{1}{\int_S G(x,y)\mathrm{d}S}$$

The expression given in Supplementary Equation 19 gives us α_{geom}, for a single sphere excitation regime.

Multi-sphere excitation factor is calculated by integration on all the spheres in the fibre, taking into account their position relative to the Gaussian beam center, as given in Supplementary equation 20:

$$(20) \quad \alpha_{\mathrm{geom}_{\mathrm{multi}}} = \frac{1}{\int_{S_0} G(x,y)\mathrm{d}S \sum_i \mathrm{e}^{-x_{\mathrm{i}}^2/2\sigma_{\mathrm{x}}^2}}$$

Here S_o is the area of the sphere that coincides with the Gaussian center, and $\sigma_x = \frac{s_{\mathrm{x}}}{2\sqrt{2\log 2}}$.

The geometric factor α_{geom} was calculated for every experiment conducted using the following experimental data: The FWHM of the beam in single sphere excitation (spherical lens) - $s_{\mathrm{x}} = s_{\mathrm{y}} = 22\mu\mathrm{m}$. The FWHM of the beam in multiple sphere excitation (cylindrical lens) $s_{\mathrm{x}} = 462\mu\mathrm{m}$; $s_{\mathrm{y}} = 180\mu\mathrm{m}$. The geometric corrections are summarized in Supplementary Table 4.

The values of the measured responsivity such as shown in Figure 5(c) and 5(d) in the main text were calculated by Supplementary equation 17, taking into account the different values of the geometric correction factors given in Supplementary Table 4. The theoretical values of the

responsivity were calculated by Supplementary equation 16, using the following summarized in Supplementary Table 5.

The values of Q_{abs} were calculated as described in Supplementary Note 4.

As a comparison between the experiments and theory, an average of the responsivity was taken in a light wavelength range of 825-850nm. Supplementary table 6 summarizes the measured responsivities and expected responsivity from theory, for one measurement from each type.

From Supplementary Table 6, we notice that the measured responsivity is very similar to the theoretical prediction, taking into to account the fact that the calculation of the theoretical responsivity was a first order model. The variance between the responsivity of the results obtained for multiple-sphere and single-sphere excitations could be attributed to the number of connected spheres. From these values it is most probable that in the multiple sphere experiment the majority of the spheres were connected in specimens with spheres of 11μm radius, and ~35% of the spheres were disconnected in specimens with 5μm radius. It is reasonable that as the sphere radius decreases, the effect of electrode fluctuations and sphere size dispersion will adversely affect the contact between the electrodes and the spheres, increasing the number of misconnections, as supported by the electrical measurements.

Supplementary Note 4 – Resonances theory

Mie theory[8] describes the interaction of electromagnetic radiation with spherical particles. The ultimate result of the theory is given by the scattering, extinction and absorption factors. We focus on the absorption as it will directly affect the absorption of the light in the semiconductor which could be measured through the response of the photodetecting spheres in the fibres.

We have measured the refractive index and the extinction coefficient of As_2Se_5 by spectrophotometry using an evaporated film (thickness of $18\mu\mathrm{m}$) of As_2Se_5 and employing Swanepoel method to calculate the refractive index of the material[11]. The results are shown in Supplementary Figure 7.

Using the values of the refractive index and extinction coefficient we obtain Q_{abs} for sphere radii of $5\mu\mathrm{m}$, $11\mu\mathrm{m}$ and $107\mu\mathrm{m}$ as shown in Supplementary Figure 8.

From Supplementary Figure 8, for smaller spheres there are resonant peaks present and the free spectral range (FSR) between these peaks get larger, as the sphere diameter is reduced, as expected from theory. These features are then measured through the photoresistive response of the fibre that contains sphere with the aforementioned radii.

Figure 5(e) was obtained by performing an FFT analysis on the calculated responsivity of fibres, while continuously changing the sphere radius (in a single sphere illumination setup). The peak location is inversely proportional to the sphere radius, as expected from conventional Fabry Perot resonator theory.

The theoretical curves, as shown in red in Figure 5(c) and 5(d) were calculated by Supplementary Equation 16. For the case of single sphere excitation, the curves were calculated by taking one sphere with the corresponding radius. For the case of multiple spheres excitation, the calculated responsivity was averaged for a random pool of spheres chosen from a Gaussian distribution with

a mean equal to the sphere radius and the standard deviation measured for each fiber type (see Supplementary Table 2). The number of samples was chosen according to Supplementary equation 21, where N_{sample} is the number of samples selected, l_{a} is the FWHM size of the spot size used to excite multiple spheres and λ is the wavelength at which the spheres appear in the fibre (Tomotika wavelength).

$$(21) \quad N_{\mathrm{sample}} = \frac{l_{\mathrm{a}}}{\lambda}$$

For spheres with a radius of $5\mu\mathrm{m}$, we have included 13 spheres in the calculation, and for spheres with a radius of $11\mu\mathrm{m}$, we have included 7 spheres in the averaging calculation of the responsivity.

This averaging leads to “smoothing” of the sharp Mie peaks, but since we are interacting with a relatively small amount of spheres, Mie peaks are still present, as can be seen in 5(d). The main FFT peak is clearly present in both cases, as can be seen in Figures 5(e) in the main text and in Supplementary Figures 9-11.

Supplementary References

1. Tomotika, S. On the Instability of a Cylindrical Thread of a Viscous Liquid Surrounded by Another Viscous Fluid. *Proc. R. Soc. A Math. Phys. Eng. Sci.* **150,** 322–337 (1935).
2. Lord Rayleigh. On the Capillary Phenomena of Jets. *Proc. R. Soc. London* **29,** 71–97 (1879).
3. Lord Rayleigh. On the instability of a cylinder of viscous liquid under capillary force. *Philos. Mag. J. Sci.* **34,** 145-154 (1892).
4. Hart, S., and Fink, Y. Interfacial energy and materials selection criteria in composite microstructured optical fiber fabrication. in *MRS Proceedings* **797,** 1–7 (2003).
5. Dann, J. Forces involved in the adhesive process. *J. Colloid Interface Sci.* **32,** 302–320 (1970).
6. Saleh B.E.A., and Teich M.C. *Fundamentals of Photonics*. Wiley Interscience, Hoboken, New Jersey (2007).
7. Marshall J.M, and Owen A.E. Drift mobility studies in vitreous arsenic triselenide. *Philosophical Magazine*, **24**, 1281-1305 (1971).
8. Bohren, C. F., and Huffman, D. R. *Absorption and scattering of light by small particles*. (Wiley, 2004).
9. Lamb D.R. *Electrical conduction mechanisms in thin insulating films*. Methuen Monographs, London, UK (1967)
10. Borisova Z.U. *Glassy semiconductors*. Plenum Press, New York, USA (1981)
11. Swanepoel R. Determination of the thickness and optical constants of amorphous silicon. *Journal of Physics E: Scientific Instruments*, **16**, 1214-1222 (1983).
